# Development and psychometric evaluation of the 12-item Polish version of the Pain Vigilance and Awareness Questionnaire

**DOI:** 10.1038/s41598-025-08449-6

**Published:** 2025-07-22

**Authors:** Joanna Kłosowska, Aleksandra Budzisz, Daryna Rubanets, Izabela Anna Łaska, Helena Bieniek, Justyna Brączyk, Karolina Wiercioch-Kuzianik, Elżbieta Anita Bajcar, Magdalena Żegleń, Magdalena Niedbał, Julia Badzińska, Anna Przeklasa-Muszyńska, Lance M. McCracken, Przemysław Bąbel

**Affiliations:** 1 Pain Research Group, Institute of Psychology, Jagiellonian University, Ingardena 6, 30-060 Krakow, Poland; 2 Doctoral School in the Social Sciences, Jagiellonian University, Krakow, Poland; 3 Department of Anthropology, Faculty of Physical Education and Sport, University of Physical Culture in Kraków, Krakow, Poland; 4 Institute of Psychology, University of the National Education Commission, Krakow, Poland; 5 Chair of Anesthesiology and Intensive Therapy, Department of Pain Research and Treatment, Jagiellonian University Medical College, Krakow, Poland; 6 Psychology Department, Uppsala University, Uppsala, Sweden

**Keywords:** Pain Vigilance and Awareness Questionnaire, Psychometric properties, Cross-cultural adaptation, Confirmatory Factor Analysis, Chronic pain, Psychology, Human behaviour, Pain

## Abstract

The Pain Vigilance and Awareness Questionnaire (PVAQ) is widely used to assess attention to pain, but no validated Polish version has been previously available. This study aimed to translate the PVAQ into Polish and evaluate its psychometric properties. Two studies were conducted: Study 1 included 418 chronic pain patients recruited online who completed the scale alongside measures of depression, anxiety, pain catastrophizing, pain anxiety, pain intensity, functional limitations, and psychological flexibility. A follow-up was completed by 79% after 30 days. Study 2 involved 148 hospital-recruited chronic pain patients who completed the adapted scale and measures of pain intensity and functional limitations. None of the previously proposed models describing the factor structure of the PVAQ scale adequately fit the data. However, factor analyses indicated that a 12-item version with two correlated factors–Passive Awareness and Active Vigilance–provided acceptable model fit. The Polish PVAQ showed good internal consistency, moderate test–retest reliability, and expected correlations with related constructs. Interestingly, Passive Awareness (but not Active Vigilance) was positively associated with components of psychological flexibility. The Polish 12-item PVAQ is reliable and valid instrument for assessing pain-related attention in chronic pain samples, aiding healthcare professionals in identifying highly vigilant individuals to tailor interventions accordingly.

## Introduction

Pain, as defined by the International Association for the Study of Pain, is “An unpleasant sensory and emotional experience associated with, or resembling that associated with, actual or potential tissue damage”^1^. This definition emphasizes not only the physical aspects of pain but also the critical role of emotion and closely related processes. In humans this implicates cognitive and attentional responses to the bodily sensations that contribute to the pain experience.

Pain automatically captures attention when it occurs. When attention is focused on the experience of pain, it may serve as a moderator of pain perception, often amplifying pain intensity and emotional impact^2–5^. This, in turn, can lead to greater emotional distress, disability, and increased use of healthcare services^6,7^.

Attention directed toward pain can be conceptualized as pain vigilance or pain awareness. Pain vigilance refers to heightened, often maladaptive focus on pain, characterized by excessive monitoring and hyperattention, which can amplify distress and impair adaptive coping. The second component, pain awareness involves recognizing and observing pain as it arises and subsides^8,9^. Its implications can be considered twofold. On the one hand, pain awareness has been shown to be positively associated with pain intensity, catastrophizing, and fear of pain^9–12^. This suggests that it may reflect heightened attention to pain, which contributes to both pain and distress. On the other hand, observing pain in non-judgmental way, as part of one’s experience, can serve as a foundation for developing pain acceptance: the ability to experience pain without attempting to control, reduce, or avoid it. Pain acceptance is an essential component of psychological flexibility^8,9,13,14^, which has been shown to protect individuals from pain-related interference^15^.

The Pain Vigilance and Awareness Questionnaire (PVAQ), developed by Lance McCracken^8^, is a self-report tool designed to assess the two distinct yet related dimensions of attention to pain highlighted above: heightened awareness of pain and increased pain vigilance. The psychometric properties of PVAQ have been assessed in several language versions, including English^8,9,16^, Dutch^10,17^, Chinese^12^, Spanish^18,19^, Swedish^20^, German^21^, Italian^11^, and Brazilian-Portuguese^22^. Validations have been performed in both non-clinical^16,17,21,22^ and clinical groups, such as those with chronic pain of various origins^8,9,11,18,19^, including chronic low back pain^8,11^, and fibromyalgia^10,20^, highlighting the scale’s utility across a spectrum of pain experiences.

Differences in the factor structure of PVAQ have emerged across language versions. Originally one-factor was hypothesized^8^, but subsequent studies have suggested that a more complex model is needed. Most language adaptations have supported a two-factor structure of PVAQ, though the specific composition of the factors varied across studies. In five studies, the obtained factors were labelled as “active vigilance” and “passive awareness”^11,12,18–20^. Four other studies identified the factors as “attention to pain” and “attention to changes in pain”^10,17,21,22^. Broadly, the first factor (active vigilance/attention to pain) corresponded to pain vigilance, while the second (passive awareness/attention to changes in pain) aligned more closely with pain awareness. Additionally, one study proposed a three-factor model comprising “awareness of change”, “intrusion” and “monitoring”^16^. These variations reflect differences in the assignment of specific items to each subscale across studies.

Adaptations of PVAQ have also resulted in modifications to its original length. While some studies retained the full 16-item version^8,16,17,21^, others proposed shortened versions, including 14-item^10^, 13-item^9,11,12,22^, 9-item^18,19^ and 8-item^20^ versions. Taken together, these findings suggest that PVAQ may be sensitive to cultural and linguistic differences.

Previous studies have established in general positive associations between PVAQ – either as an overall score or through specific subscales – and various pain-related variables, including pain intensity, depression, anxiety, fear of pain, disability, pain catastrophizing, pain acceptance, and kinesiophobia^10–12,16–21,23^.The relationship between PVAQ and depression has been assessed using the Beck Depression Inventory^8^ and the Hospital Anxiety and Depression Scale (HADS)^11,12,18,19,21^. Observed correlations were low and positive, ranging from r = 0.20^19^ to r = 0.28^11^. For anxiety, as measured by HADS, one study found no significant relationship^21^, while others reported low to moderate positive correlations, ranging from r = 0.22 to r = 0.53^11,12^.

Pain catastrophizing has been evaluated using the Pain Catastrophizing Scale (PCS), with studies reporting strong positive correlations with PVAQ (r = 0.68)^10,17^.

Similarly, pain-related anxiety, as measured by the Pain Anxiety Symptoms Scale (PASS), has shown strong positive correlations with PVAQ, ranging from r = 0.50 to r = 0.61^8,10,19,21^. The PVAQ attention to pain subscale has demonstrated even higher correlations, ranging from r = 0.66 to r = 0.70^10,21^.

Disability in pain populations has been shown to correlate positively with PVAQ scores, with correlations ranging from weak to strong (r = 0.13 to r = 0.88)^11,12,20^. Similarly, pain intensity has demonstrated a positive relationship with PVAQ scores, with correlations ranging from r = 0.17 to r = 0.88, depending on the study^12,16,21^.

The PVAQ has demonstrated acceptable to good internal consistency across studies, with Cronbach’s alphas for total scores ranging from 0.75^12^ to 0.92^16^. For the subscales, Cronbach’s alphas varied from 0.72^21^ to 0.92^18^. The test–retest reliability of the Italian version of PVAQ has been evaluated over a 10-day interval, in which its stability was confirmed. Intraclass Correlation Coefficient (ICC) ranged from 0.88 to 0.92 (depending on the score), indicating high reliability^11^.

Based on these findings, it is anticipated that the Polish version of PVAQ will exhibit good internal consistency and reliability. Significant correlations are hypothesized between PVAQ scores and several pain-related constructs and measures of psychological well-being, including weak positive correlations with depression, weak to moderate positive correlation with anxiety, strong positive correlations with pain catastrophizing, strong positive correlations with pain anxiety, and positive correlation with pain intensity. Additionally, given the consistently positive correlations between PVAQ scores and pain-related disability reported in previous studies^11,12,20^, it is anticipated that PVAQ scores will vary based on self-reported functional limitation due to pain, such as being on sick leave, ceasing work or academic activities due to pain, and withdrawing from important life activities as a result of pain. Finally, building on theoretical considerations^14,24–26^, we hypothesize that pain awareness but not pain vigilance will show positive correlations with facets of psychological flexibility, such as self-as-context (ability to take a perspective from which one observes thoughts and emotions without being entirely defined by them^14^) and committed action (engagement in valued activities despite pain^14^).

Given that previous validation studies of the PVAQ have yielded various factor structures, we did not hypothesize a single specific factor structure a priori. Instead, we planned to test several previously reported structures using Confirmatory Factor Analysis to evaluate their fit in the Polish sample. If none of the previously reported models demonstrated acceptable fit, we intended to conduct an exploratory factor analysis to further examine the underlying structure.

## Study 1

The aim of Study 1, conducted with a relatively large sample of individuals experiencing chronic pain, was to develop a Polish version of PVAQ and to evaluate the structure and psychometric properties of the translation.

### Results

#### Preliminary analyses

All PVAQ items exhibited skewness and kurtosis values from – 1 to 1, indicating approximately normal univariate distributions. However, Mardia’s test revealed a significant deviation from multivariate normality (χ^2^ = 2415.49, *df* = 816, *p* < 0.001, multivariate kurtosis: *z* = 33.36, *p* < 0.001). Consequently, an appropriate estimator was selected to account for these deviations. Descriptive statistics for each item are presented in the supplementary materials (Supplementary Table 1).

#### Structural validity

The tested models that were obtained in previous studies (see: Supplementary Table 2 for details) did not demonstrate an acceptable fit. Modification Indices (MIs) indicated shared covariance among several pairs of item errors across the models. Specifically, Model 1, which exhibited the poorest fit, identified 25 error pairs with MI values ≥ 15. In contrast, Model 8, which showed the best relative fit, had 5 error pairs with MI values ≥ 15. Importantly, many of these correlations involved items from different scales. Even in the best-fitting model (Model 8), incorporating at least five additional covariances was necessary to achieve a reasonable fit.

**Fig. 1:**
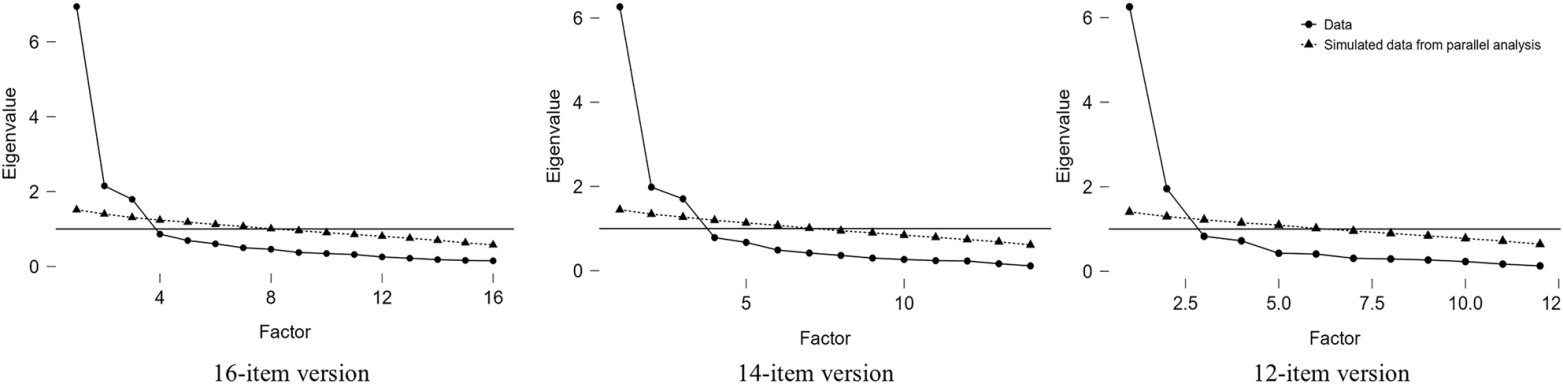
Scree plots for exploratory factor analyses-Study 1. N = 209; The two-factor model including 12 items was retained.

These findings suggest that the factor structure of the Polish version of PVAQ may differ from previously established models. Consequently, we randomly divided the sample in half and conducted an exploratory factor analysis (EFA) on the first half (*N* = 209). The Kaiser–Meyer–Olkin statistic was 0.85, indicating good suitability of the data for factor analysis. Principal axis factoring with Promax rotation, performed on polychronic correlations, preceded by parallel analysis and visual inspection of the scree plot (Fig. 1), revealed a three-factor solution explaining 61% of variance. Items 1 and 10 exhibited a low factor loading (< 0.50) and cross-loaded equally on two factors, leading to their removal (resulting in 14 items total). The EFA that followed showed a three-factor solution explaining 63% variance (Supplementary Table 3).

Next, we examined item-rest correlations (IRC) and item reliability statistics to eliminate redundant items and items that were not well-aligned with the broader construct that the overall scale was intended to measure. Except for two items (item 8: IRC = 0.11, item 16: IRC = -0.04), all IRCs fell in the range 0.30–0.69 (see: Supplementary Table 4). Therefore, we excluded items 8 and 16 resulting in 12 items. After running EFA once more, a two-factor solution was revealed that explained 63% variance.

**Fig. 2:**
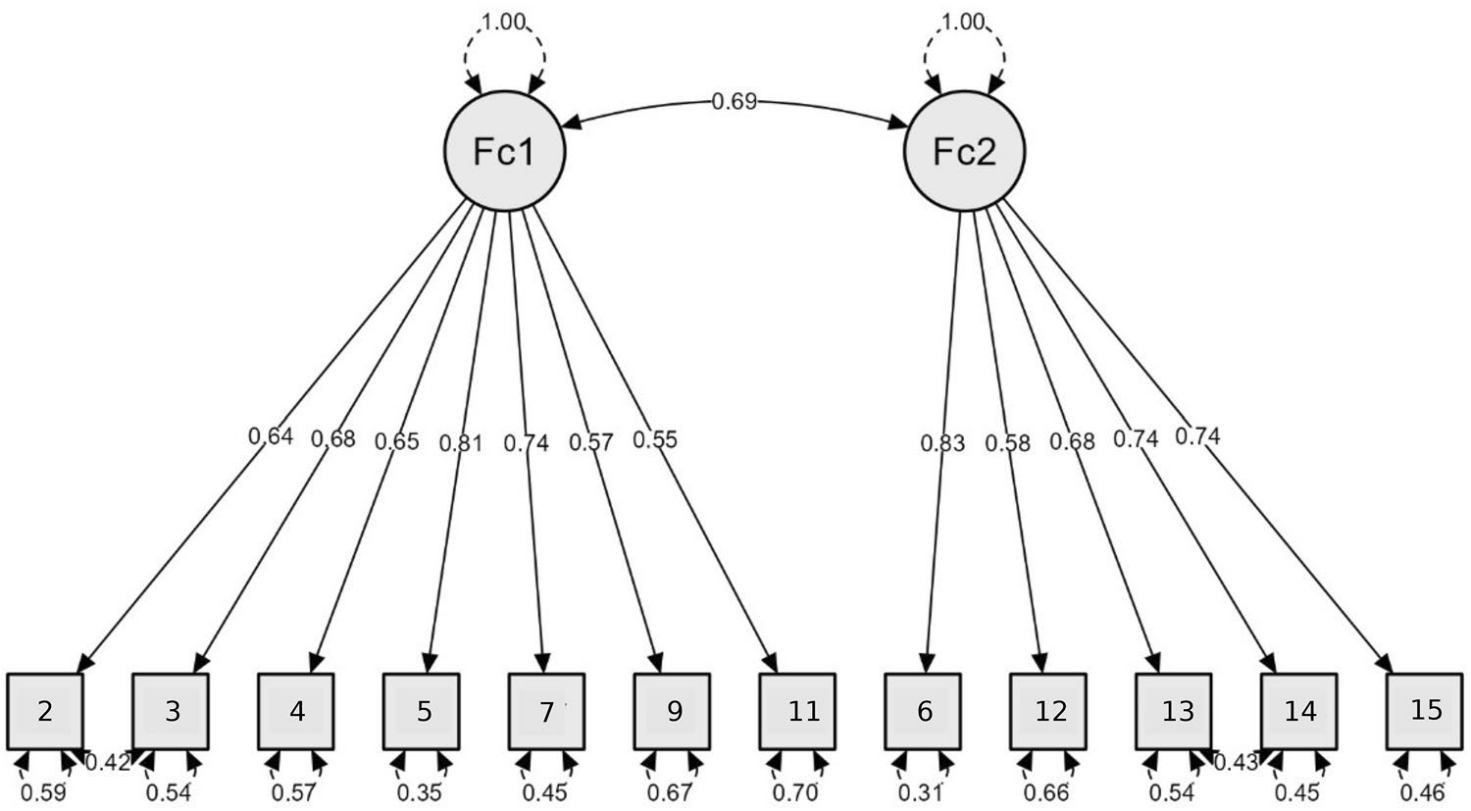
Results of Confirmatory Factor Analysis–Study 1. N = 209; *Fc1* Passive Awareness, *Fc2* Active Vigilance; Standardized coefficients are presented.

We subsequently performed Confirmatory Factor Analysis (CFA) on the second half of the sample (*N* = 209) to test the obtained structure of the 12-item version of PVAQ (PVAQ-12). Although Comparative Fit Index (CFI), Normed Fit Index (NFI), chi square/df ratio (*χ*^*2*^*/df* ) fell in a reasonable range, the Root Mean Square Error of Approximation (RMSEA) did not indicate acceptable fit (*CFI* = 0.98, *NFI* = 0.97, *RMSEA* = 0.097, 90% *CI* 0.08–0.11, *χ*^*2*^*/df* = 3.05). MIs revealed correlated residuals between items 2 and 3, as well as between 13 and 14. These error pairs consistently emerged as the top suggested modifications in six (item 2 and 3) and four (13 and 14) of the eight tested models from the previous studies. These items are positioned next to each other within the questionnaire and assess similar concepts (e.g., item 2, “*I am aware of quick changes in pain*”, and item 3, “*I am quick to notice when pain goes up or down*”), thus justifying adding these covariates to the model. The final model exhibited acceptable to good descriptive fit, depending on the index (*CFI* = 0.99, *NFI* = 0.98, *RMSEA* = 0.075, 90% *CI* 0.06–0.09, χ^*2*^*/df* = 2.22) (see Fig. 2 and Supplementary Table 5). The full list of the 12 items comprising the Polish PVAQ is included in the Supplementary Materials.

#### Reliability and items properties

In the first measurement, 0% participants recorded the lowest or highest possible scores on the total PVAQ. For the Passive Awareness subscale, 0% obtained the minimum score, while 5.7% reached the maximum. The Active Vigilance subscale had 0.2% at the minimum and 3.6% at the maximum. In the second measurement, 0.2% scored at the minimum and 0% at the maximum on the total scale. For Passive Awareness, 0.5% scored at the minimum and 3.6% at the maximum, while for Active Vigilance, 1% and 2.4% reached the minimum and maximum scores, respectively. These results indicate no floor or ceiling effects.

McDonald’s omega values (calculated for the total sample: N = 418) indicated good to excellent reliability of the Polish PVAQ-12 subscales: Passive Awareness *ω* = 0.88, 95% *CI* 0.86–0.90, Active Vigilance *ω* = 0.88, 95% *CI* 0.86–0.90 and for the total scale *ω* = 0.90, 95% *CI* 0.88–0.91. The item-rest correlations were all > 0.50 (details in Supplementary Table 6).

The Intraclass Correlation (ICC) analysis indicated that the test–retest reliability of the total PVAQ-12 score was 0.63, 95% *CI* 0.56–0.70 (p < 0.001). The subscales demonstrated the following ICC values: 0.63, 95% *CI* 0.54–0.70 for the Passive Awareness subscale and 0.63, 95% *CI* 0.56–0.69 for the Active Vigilance subscale. These results suggest that the Polish PVAQ-12 demonstrates moderate stability over time (30-day interval) across all subscales.

The Minimal Detectable Change (MDC95%) for the PVAQ-12 total score was 16.30, signifying that a change of at least 17 points in the questionnaire is necessary to denote an actual change rather than simply a measurement error. The MDC95% values for the subscales were: 9.62 for Passive Awareness and 9.07 for Active Vigilance.

#### Construct validity

Correlation analysis indicated a strong positive relationship between the PVAQ-12 total score and general pain anxiety (r = 0.55, p < 0.001). Additionally, there was a moderate positive correlation with pain catastrophizing (r = 0.39, p < 0.001). Positive but weak correlations were observed between PVAQ-12 total and depression, anxiety, pain intensity and self-as-context.

The Passive Awareness subscale demonstrated weak positive correlations with pain intensity (both at the time of the study, *r* = 0.15, *p* = 0.002, and during the previous week, *r* = 0.29, *p* < 0.001), pain catastrophizing (*r* = 0.18, *p* < 0.001) and committed action (r = 0.20, p < 0.001). Additionally, a moderate positive correlation was observed with pain anxiety (*r* = 0.35, *p* < 0.001) as well as self-as-context (r = 0.33, p < 0.001).

The Active Vigilance subscale of PVAQ-12 showed weak positive correlations with pain intensity (at the time of the study, *r* = 0.23, *p* < 0.001; in the previous week, *r* = 0.20, *p* < 0.001), and depression (r = 0.20, p < 0.001), and a very weak negative correlation with committed action (r = – 0.13, p = 0.007). Furthermore, it exhibited a moderate positive correlation with anxiety (*r* = 0.30, *p* < 0.001). Strong positive correlations with pain catastrophizing (*r* = 0.51, *p* < 0.001) and general pain anxiety (*r* = 0.60, *p* < 0.001) were observed (see Supplementary Table 7).

There were significant differences between participants who had to give up work or school due to pain and those who did not, with the former group reporting higher PVAQ-12 scores (Total: *t*(416) = 3.63, *p* < 0.001, *d* = 0.36, Passive Awareness: *t*(416) = 2.96, *p* = 0.004, *d* = 0.29, Active Vigilance: *t*(416) = 3.40, *p* < 0.001, *d* = 0.34). Individuals who reported giving up activities important to them due to chronic pain had higher PVAQ-12 total scores (*t*(416) = 3.54, *p* = 0.002, *d* = 0.42) and scored higher on the Passive Awareness subscale (*t(*416) = 3.50, *p* = 0.001, *d* = 0.42). No significant differences were found on the Active Vigilance subscale (*t*(416) = 2.66, *p* = 0.009, *d* = 0.32). Participants who reported being on sick leave did not differ in PVAQ-12 scores from those who were not (Total: *t*(260) = 0.94, *p* = 0.357, *d* = 0.15, Passive Awareness: *t*(260) = 0.29, *p* = 0.763, *d* = 0.05, Active Vigilance: *t*(260) = 2.01, *p* = 0.040, *d* = 0.32).

## Study 2

The aim of Study 2 was to verify the structure of the Polish Pain Awareness and Vigilance Questionnaire identified in Study 1, this time using a clinical sample of patients with chronic pain recruited from a hospital.

### Results

#### Preliminary analyses

Mardia’s test indicated a significant deviation from multivariate normality, with a skewness chi-square statistic of 916.87 *(df* = 364, *p* < 0.001) and a multivariate kurtosis z-score of 11.06 (*p* < 0.001). There was no evidence of ceiling or floor effects: 0% of participants obtained the minimum, and 1.50% obtained the maximum total score on PVAQ-12. For Passive Awareness, 0.80% of participants obtained the minimum score and 4.50% obtained the maximum score; for Active Vigilance, 0.70% obtained the minimum score and 5.90% obtained the maximum score. Descriptive statistics for the items and subscales are in the Supplementary Table 8.

#### Structural validity

**Fig. 3:**
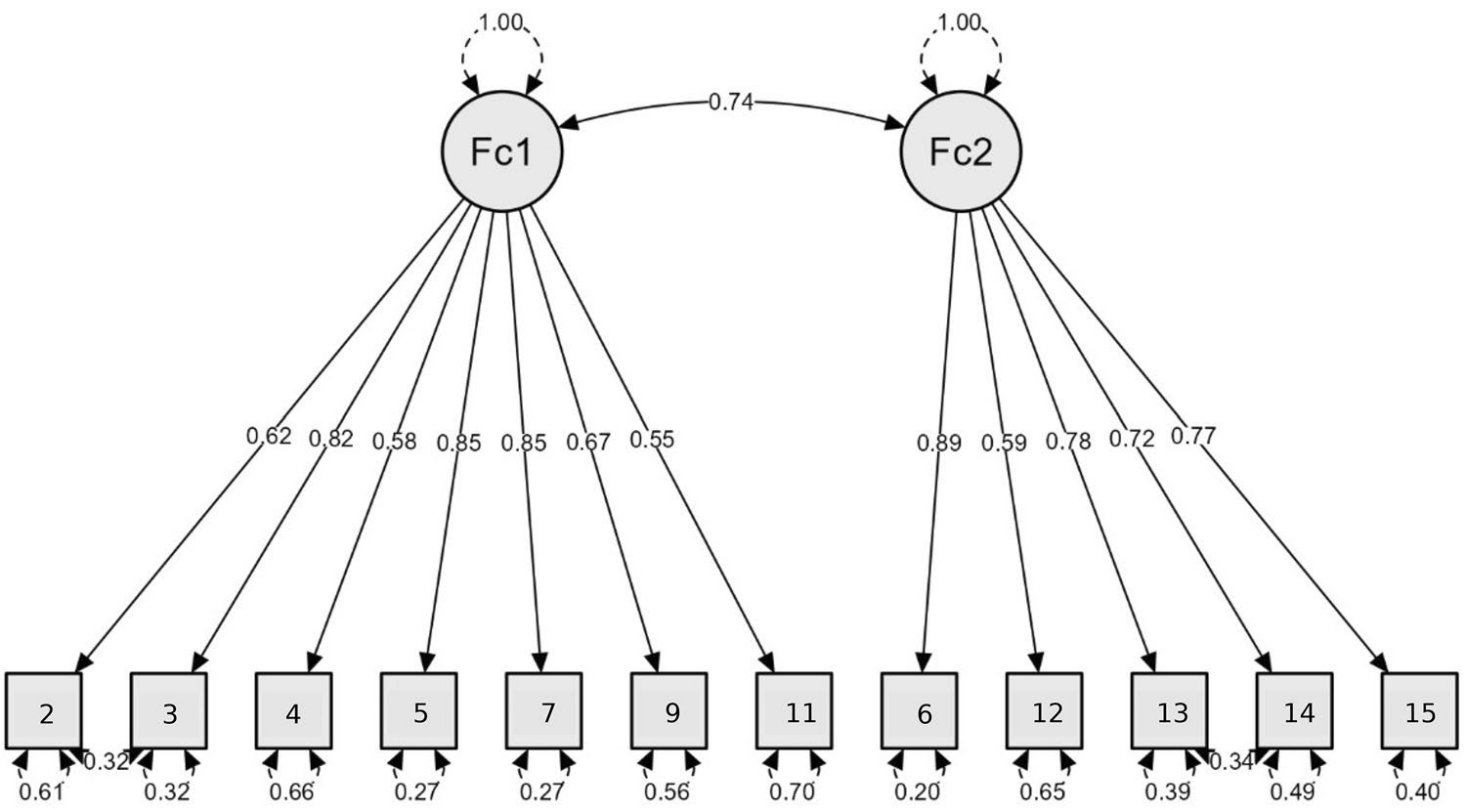
Results of Confirmatory Factor Analysis–Study 2. N = 131 (cases with missing data were deleted listwise); *Fc1* Passive Awareness, *Fc2* Active Vigilance; Standardized coefficients are presented.

CFA was performed to evaluate the two-factor structure of PVAQ-12 obtained in Study 1. Fit indices showed the good fit of this model, indicating that it describes the structure of the Polish version of the scale very well: *CFI* = 0.99, *NFI* = 0.98, *RMSEA* = 0.063, 90% *CI* 0.03–0.09, *χ*^*2*^*/df* = 1.53. The items showed high and significant factor loadings, ranging from 0.55 to 0.89 (Fig. 3).

#### Reliability and item properties

The Polish version of PVAQ-12 demonstrated good internal consistency: *ω* = 0.84, 95% *CI* 0.76–0.90 for the Passive Awareness subscale, *ω* = 0.84, 95% *CI* 0.79–0.88 for the Active Vigilance subscale, and *ω* = 0.88, 95% *CI* 0.83–0.92 for the total score. All item-rest correlations were > 0.45 (Supplementary Table 9).

#### Construct validity

The total PVAQ-12 score showed a significant positive correlation with pain intensity, both at the time of the study (*r* = 0.29, *p* < 0.001) and during the previous week (*r* = 0.33, *p* < 0.001). Similarly, the Active Vigilance subscale demonstrated significant moderate positive correlations with pain intensity (at the time of the study: *r* = 0.31, *p* < 0.001; during the previous week: *r* = 0.35, *p* < 0.001). The Passive Awareness subscale correlated positively but weakly with average pain intensity during the previous week (*r* = 0.25, *p* = 0.005) but not with pain intensity at the time of the study (*r* = 0.21, *p* = 0.028).

Participants who had given up work and/or school due to pain did not differ in PVAQ-12 scores from those who had not (Total: *t(*124) = 2.02, *p* = 0.054, *d* = 0.36, Passive Awareness: *t(*124) = 1.27, *p* = 0.213, *d* = 0.23, Active Vigilance: *t*(124) = 2.29, *p* = 0.024, *d* = 0.43). Similarly, no significant difference was observed between patients who reported being on sick leave and those who did not (Total: *t*(118) = 0.84, *p* = 0.84, *d* = 0.06, Passive Awareness: *t*(118) = 0.74, *p* = 0.462, *d* = 0.22, Active Vigilance: *t*(118) = 0.43, *p* = 0.672, *d* = 0.13), and between those who had had to resign from activities important to them and those who had not (Total: *t*(128) = 2.49, *p* = 0.019, *d* = 0.63, Passive Awareness: *t*(128) = 2.49, *p* = 0.032, *d* = 0.63, Active Vigilance: *t*(128) = 1.92, *p* = 0.042, *d* = 0.49).

### Discussion

The current project aimed to adapt PVAQ into Polish and evaluate its structure, item properties, reliability, and validity. The findings from the two studies provided support for the acceptable psychometric properties of the Polish translation. Analyses supported a two-factor structure of PVAQ, retaining 12 items. Additionally, the scale exhibited good internal consistency, robust factor loadings, and moderate temporal stability over a one-month interval. Construct validity was supported by significant positive correlations with the PASS total score, CSQ Catastrophizing score, DASS-21 Depression and Anxiety scores, and pain measures. Additionally, the two identified factors – Passive Awareness and Active Vigilance – exhibited some distinct correlation patterns with the scales measuring components of psychological flexibility (CAQ, SEQ-8), supporting construct differentiation. Associations with self-reported functional limitations due to pain were evident in the online sample but not in the hospital-recruited sample.

#### Reliability

In Study 1, the Polish PVAQ revealed excellent internal consistency, with McDonald’s omega values of 0.90 for the full scale and 0.88 for the subscales. Similarly, in Study 2 internal consistency was excellent (0.88) for the total score and good (0.84) for the two subscales. These findings align with previous research^11,12,18–20^. Additionally, high item-rest correlations (> 0.50 in Study 1 and > 0.45 in Study 2) indicated strong item homogeneity.

The stability of the Polish PVAQ over time was examined using the test–retest method, revealing moderate stability across all subscales (total = 0.63). This value was lower than in a previous study^11^, which had been expected considering the three times longer time interval applied in the current project. The MDC95% values indicate that a minimum change of 17 points in the PVAQ-12 total score is required to reflect true changes in individuals’ pain-related attention and awareness after 1 month.

#### Construct validity

The hypothesized associations between PVAQ and related constructs were largely confirmed. Additionally, distinct correlation patterns were observed for the subscores, thus supporting the division of the Polish PVAQ-12 into two distinct scales: Passive Awareness and Active Vigilance.

Average pain intensity scores showed weak to moderate positive correlations with the PVAQ-12 total score and its subscales, mirroring findings from several validation studies^9,11,20,21^. In contrast, the Chinese version of PVAQ reported a strong correlation between the Passive Awareness subscale and pain intensity (r = 0.88^12^). However, it is worth noting that Wong et al.^12^ applied a broader definition of pain intensity, calculating it as the mean of current, average, and worst pain ratings. This composite approach may provide a more accurate estimate of overall pain experience, potentially reducing measurement error and resulting in stronger associations with cognitive factors such as attention to pain. Future research should therefore clarify how different approaches to pain measurement influence the relationship between attention to pain and pain intensity across various cultural contexts.

As is consistent with our hypothesis, individuals with higher and lower levels of functional limitations due to pain, differed significantly in the PVAQ scores. In the online sample, participants who had quit work or studies due to pain scored higher on PVAQ in comparison to those who had not. Moreover, participants who indicated forgoing important activities because of pain scored higher on both the total PVAQ and the Passive Awareness subscale. However, these differences were not observed in the hospital-recruited sample, likely due to the more homogeneous nature of the clinical group, which was characterized by high levels of disability.

The total PVAQ score demonstrated only a moderate (not strong as hypothesized) positive correlation with pain catastrophizing. However, correlation patterns varied across subscales: the Active Vigilance subscale showed a strong correlation, while the Passive Awareness subscale showed only a weak one. This finding is theoretically consistent, given that pain catastrophizing – characterized by an exaggerated perception of threat, feelings of helplessness, and difficulty suppressing pain-related thoughts^27^ – is closely linked to vigilant attention to pain. Moreover, these constructs appear to influence each other: ongoing worry about chronic pain may sustain heightened vigilance, perpetuating the perception of threat^28^.

The hypothesized strong correlations between the PVAQ scores and the measure of pain anxiety were largely confirmed. The PVAQ-12 total score exhibited a strong positive correlation with pain anxiety, with even higher correlations observed for the Active Vigilance subscale. In contrast, the Passive Awareness subscale demonstrated only a moderate association. These results are consistent with previous studies^9,10,18,19,21^, highlighting the capacity of the Active Vigilance subscale to effectively measure heightened sensitivity to anxiety-driven pain responses. This suggests that the Active Vigilance dimension is closely related to pain anxiety, reflecting a strong conceptual and empirical link between these constructs. These findings support the utility of PVAQ in identifying hypervigilant, anxiety-driven pain patterns.

Anxiety was weakly and positively correlated with the total PVAQ-12 score and moderately with the Active Vigilance subscore. Additionally, weak positive correlations were observed between depression and both the PVAQ-12 total score and the Active Vigilance subscale. These findings are consistent with earlier research^11,12,19^ and show that while anxiety, depression, and pain attention are interrelated, they likely represent distinct constructs.

In the current study, the Passive Awareness and Active Vigilance subscales appeared to demonstrate distinct associations with pain-related variables. Active Vigilance showed strong links to maladaptive constructs such as pain anxiety and catastrophizing, and it correlated significantly with psychopathological symptoms like anxiety and depression. In contrast, Passive Awareness demonstrated weaker associations with pain anxiety and catastrophizing and did not significantly correlate with depression or anxiety symptoms, thus supporting the findings of some previous studies (e.g.,^9^).

Further differences emerged in relation to psychological flexibility. Passive Awareness was positively associated with two components of psychological flexibility – committed action and self-as-context – whereas Active Vigilance either showed no relationship or negative correlations with these constructs. These patterns align with theoretical models distinguishing between constructive awareness and maladaptive vigilance, suggesting that different forms of attentional focus on pain may play distinct roles in adaptation to chronic pain^9,13,14,24^.

The current findings can be better understood within the broader framework of psychological flexibility, a concept central to Acceptance and Commitment Therapy (ACT). Psychological flexibility refers to the ability to stay aware of thoughts, emotions, and bodily sensations–such as pain–while choosing behaviors aligned with one’s values and recognizing as well as adapting to situational demands^24^. ACT-based interventions aim to cultivate these skills and have demonstrated considerable effectiveness in improving well-being in individuals with chronic pain^29–34^.

Psychological flexibility is built on six core skills: present-focused awareness, acceptance, cognitive defusion, self-as-context, value-based action, and committed action^14^. Among these, committed action involves goal-directed behavior that is both persistent (allowing individuals to continue despite setbacks or discomfort) and flexible (enabling adjustments when necessary). Within ACT, committed action fosters engagement in valued activities despite pain, contributing to better mental health and overall functioning^24^. Similarly, self-as-context is a crucial aspect of psychological flexibility that refers to the ability to take a perspective from which one observes thoughts and emotions without being entirely defined by them. Improvements in self-as-context, particularly through ACT, are associated with enhanced psychological functioning, as individuals become less entangled in distressing self-evaluations and more capable of adaptive, values-driven action^25,26^.

From this perspective, the Active Vigilance – as measured by PVAQ appears to reflect a rigid and threat-driven monitoring of pain, potentially motivated by fear or perceived danger. Such hypervigilance may interfere with flexible functioning and engagement in values-based actions. In contrast, Passive Awareness may represent a more observational mode of attention to changes in pain. While this heightened focus can be associated with increased pain intensity and distress—especially when accompanied by efforts to control or avoid pain—it may also reflect a capacity to notice pain without immediately reacting to it. In this sense, awareness encompasses dual possibilities: one can be aware-and-resist or aware-and-allow^9^. Individuals who relate to their pain in a more receptive and non-reactive way may be better equipped to sustain goal-directed behavior and maintain a broader, more stable sense of self that is not wholly defined by the pain experience.

Taken together, these results suggest that Passive Awareness as measured by the PVAQ may represent a more nuanced–and potentially less maladaptive–form of pain attention than Active Vigilance in chronic pain contexts, thus highlighting the importance of using these distinct subscales for clinical assessment. Furthermore, Passive Awareness may itself encompass distinct subtypes, some of which may be more adaptive than others depending on the context- such as whether it is accompanied by acceptance–and on how pain influences emotion and behavior. This complexity may explain why Passive Awareness is positively associated with both pain-related distress and facets of psychological flexibility. The current version of the PVAQ does not address this potential distinction, which may be a valuable avenue for future research.

#### Structural validity

There is no consensus regarding the structure of PVAQ, as different language adaptations have resulted in varying subscales and different numbers of items^9,10,16^. This suggests that the scale may be influenced by cultural and language-related factors.

In the current study, CFAs were conducted to assess the fit of several previously identified PVAQ structures. As none of these models achieved acceptable fit, an EFA was performed to explore the structure of the Polish PVAQ. This analysis identified a two-factor structure with 12 items, which demonstrated acceptable to good fit and high factor loadings in subsequent CFAs performed on both online and hospital-recruited samples. The resulting structure aligns with previous adaptation studies^9,11,12,18–20^ (a comparison of the factor structures identified in this study and those proposed in previous research is presented in Supplementary Table 10).

To achieve an acceptable fit for the two-factor model in the CFA, correlations were introduced between items 2–3 and 13–14. Items 2 and 3 both refer to awareness of changes in pain intensity, while items 13 and 14 reflect a constant, background monitoring of pain sensations. In addition to their conceptual similarity, each item pair also shares highly similar wording and appears consecutively in the questionnaire, which may contribute to shared variance due to factors such as context effects–where respondents draw on information activated by one item when answering the next^35^. Omitting correlated residuals in such cases may lead to model misspecification^35^. It is also worth noting that the inclusion of residual covariances between item pairs (in some cases up to four) has been reported in previous language adaptations of the PVAQ^11,22^.

Four items (1, 8, 11, and 16) were excluded from the Polish version of PVAQ due to low factor loadings and weak item-rest correlations. Notably, items 8 and 16 are reverse scaled, which may lead to participant confusion and difficulties in answering these questions^22^. Furthermore, these two items have frequently been excluded in prior studies evaluating the psychometric properties of PVAQ^9–12,18–20,22^. Items 1 and 11 have also been removed in some language adaptations; for instance, both were excluded in the Swedish validation study^20^, while item 1 was additionally removed in the Spanish and Brazilian Portuguese adaptations^18,22^. It is worth mentioning that the translations of the full 16-item PVAQ are generally not regarded as psychometrically sound^20^, and shorter versions are considered more practical in clinical settings. Taken together, these findings support the decision to shorten the Polish version of the scale.

#### Limitations

Some limitations of the study should be acknowledged. In Study 1, self-reported chronic pain diagnoses were not confirmed through clinicians’ assessments. In contrast, Study 2 involved hospital patients undergoing chronic pain treatment, ensuring clinically verified diagnoses. Additionally, the study assessed only convergent validity, without examining divergent validity. However, this was also the case for most of the previous PVAQ adaptations^11,12,18,20,21^. Future research should address these limitations and further explore how PVAQ-12 scores correlate with treatment progress or improvements in patients’ functionality in order to establish their predictive value and utility in clinical settings.

#### Conclusions

The Polish 12-item version of PVAQ demonstrated good psychometric properties in chronic pain samples. Its two-factor structure aligns with previous research, including the English version^9^. A strength of this adaptation study is the rigorous translation process and the evaluation of psychometric properties in two independent samples comprising both the community sample and patients. The study provides additional evidence regarding the scale’s stability over a one-month interval, thus addressing a gap in the literature as previous assessments were usually limited to shorter intervals. Furthermore, the findings reinforce the measure’s validity by revealing positive associations between Passive Awareness (but not Active Vigilance) and components of psychological flexibility, highlighting the differentiation of the two facets of attention to pain. Overall, the results enhance our understanding of PVAQ’s applicability in both research and clinical settings.

Internationally, PVAQ is widely used^8,10–12,16–22^ as a valid measurement of pain vigilance and awareness. However, it has not been available for Polish-speaking populations. This translated and evaluated 12-item version of PVAQ is a valuable tool for Polish healthcare professionals and researchers that provides a resource for monitoring adaptation to chronic pain and treatment progress, especially in the context of the growing popularity of ACT. Additionally, this tool may encourage further cross-cultural research into the role of pain-related attention in chronic conditions.

## Methods

### Study 1

#### Participants

The minimum sample size required to test the hypothesized structure of PVAQ was determined a priori based on the criterion proposed by Bentler and Chou^36^: a minimum ratio of 5:1 cases per estimated parameter in the structural equation model. For the most complex structure identified in previous studies, which involved three latent variables and 16 items (resulting in 35 parameters to be estimated), a minimum of 175 participants reporting chronic pain was deemed necessary to perform CFA, assuming high factor loadings and multiple indicators per latent variable. Given that the project aimed to validate multiple scales (see: Measures), the final sample included 418 participants. The study recruited participants from “ARIADNA”—a large Polish research panel. Before the study, an initial survey was conducted to identify individuals with chronic pain, revealing that 22% of panelists met this criterion, with 53% having a physician’s diagnosis. To participate, individuals had to be over 18 years of age, fluent in Polish, have pain lasting over 3 months, and have a physician’s diagnosis of chronic pain. Of the 15,760 invited panelists, 2569 completed the survey, with 418 meeting all inclusion criteria. The final sample included 168 men (40.19%), 249 women (59.57%), and one non-binary individual (0.24%); age range was 19–86 years (M = 49.37, SD = 15.20). Participants completed the survey twice to evaluate test–retest reliability. A total of 330 participants completed both measurements, conducted one month apart, including 144 men (85.71%) and 186 women (74.70%) aged 19–84 (M = 51.21, SD = 14.79). The sociodemographic and pain characteristics of the participants are described in detail in Kłosowska et al.^37^.

#### Measures

Participants completed an online survey which included the following: (1) demographic information (sex, age, employment status, income, marital status, education, and residence); (2) chronic pain details (diagnosis, duration, location, intensity); (3) medical care history (current and past treatments for chronic pain, including duration, and type of treatment); and (4) functional limitations due to pain (being on sick leave, leaving work or university, or limiting important activities due to pain).

The survey included binary, multiple-choice, and open-ended questions. Average pain intensity (at the time of the study and in the previous week) was assessed using an 11-point numerical rating scale, where 0 indicated no pain and 10 represented the worst pain imaginable.

After the survey, participants also filled out the following questionnaires:

- *The Pain Vigilance and Awareness Questionnaire (PVAQ)*^8^ is a measure of attention to pain that is applicable to a variety of pain populations. PVAQ contains 16 items rated on a 6-point Likert scale from 0 (Never) to 5 (Always). The Pain Vigilance and Awareness Questionnaire has a Cronbach’s alpha of 0.86 for the English version^8^.
- *The Depression Anxiety Stress Scales 21 (DASS-21)*^38^; Polish adaptation^39^ of the DASS consists of 21 items designed to measure three closely related negative emotional states: (1) depression, (2) anxiety, and (3) stress. Respondents rate each item on a 4-point Likert scale, ranging from 0 ('Did not apply to me at all/never’) to 3 ('Applied to me very much, or most of the time/almost always’). Since previous validation studies have not examined the relationship between stress symptoms and PVAQ scores–limiting our ability to formulate clear hypotheses regarding this association–for the current study, only the depression and anxiety subscales were used to assess the validity of the PVAQ. The Polish adaptation of the DASS-21 demonstrates excellent internal consistency with a Cronbach’s alpha of 0.93 for the total score ^39^. In the current sample McDonald’s omega for the total score: 0.96, for the depression subscale: 0.92, for the anxiety subscale: 0.90.
- *The Catastrophizing Scale from the Coping Strategies Questionnaire (CSQ)*^40^; Polish adaptation^41^ consists of six items designed to assess catastrophizing as a cognitive strategy for managing pain. Each item is scored from 0 (Never) to 6 (Always). The Catastrophizing Scale has Cronbach’s alpha of 0.80 for the total score in the Polish adaptation^41^. In the current sample McDonald’s omega for this subscale was 0.88.
- *The Pain Anxiety Symptoms Scale 20 (PASS-20)*^42^; is a 20-item questionnaire measuring pain-related anxiety. Polish adaptation^37^ of the PASS-20 consists of four subscales: physiological responses, cognitive anxiety, fearful appraisal of pain, escape and avoidance behaviors. Total score reflecting general pain anxiety can also be calculated. Each item is scored on a scale from 0 (Never) to 5 (Always). Cronbach’s alpha for the total score in the Polish adaptation, based on the current sample, was 0.96 (McDonald’s omega also = 0.96).
- *CAQ-8 The Committed Action Questionnaire (CAQ-8)*^43^; Polish adaptation^44^ is a validated shorter version of the 18-item Committed Action Questionnaire (CAQ). This 8-item self-report instrument is intended to measure committed action – a facet of psychological flexibility. Each item assesses goal-directed behavior and flexibility in the face of difficulties. Respondents are asked to rate the extent to which each item applies to them on a Likert scale from 0 (Never True) to 6 (Always True). CAQ-8 has a Cronbach’s alpha of 0.84 for the total score in the Polish adaptation^44^. In the current study McDonald’s omega was 0.74.
- *Self-Experiences Questionnaire (SEQ)*^25^; Polish adaptation^44^ is a 15-item questionnaire for measuring “self-as-context” in two aspects: self as a distinction (the ability to differentiate oneself from thoughts and feelings) and self as an observer (a sense of self as observer of one’s experiences). Each item is scored on a scale from 0 (Never true) to 6 (Always true). Cronbach’s alpha for the Polish adaptation is 0.91 for the self as a distinction subscale, and 0.89 for the self as an observer subscale^44^. In the current study McDonald’s omega was 0.89 for the self as a distinction subscale and 0.92 for the self as an observer subscale.

As part of the larger project, several additional tools were employed, though they are not detailed in this section. These include the Chronic Pain Acceptance Questionnaire–Revised (CPAQ-R)^13^, the Chronic Pain Values Inventory (CPVI)^45^, the Pain Sensitivity Questionnaire (PSQ)^46^; Polish version:^47^, and the State-Trait Anxiety Inventory (STAI)^48^; Polish adaptation:^49^.

#### Procedure

The Polish translation of the questionnaire followed the ISPOR Task Force for Translation and Cultural Adaptation guidelines^50^. Approval was obtained from Professor McCracken, who also offered guidance on the translation and study design. A forward–backward translation approach was used: two professional translators created independent Polish versions, which were then merged into a single consensus translation. An independent translator subsequently produced a back-translation into English, reviewed by the project principal investigator and a native English speaker. A harmonization meeting compared all versions against each other and the original source. Ten Polish-speaking individuals participated in cognitive testing, and the translation was finalized based on their feedback. To ensure broader applicability, both pain-free individuals and participants suffering from chronic pain (50% each) were included in the debriefing phase. This approach is consistent with previous research demonstrating that the PVAQ is valid for use in both pain-free and clinical populations^21^, and it acknowledges the potential for the Polish version to be applied in a wider range of populations in future studies.

Data collection occurred via an online survey platform. Participants first viewed information about the study’s aims and were informed that participation was voluntary and anonymous. Informed consent was obtained by having them select the appropriate option. They then completed a 45-min set of questionnaires (PVAQ, CPAQ-R, PASS-20, CPVI, CSQ, PSQ, STAI-T, DASS-21, CAQ-8, and SEQ), plus a sociodemographic and illness history form. After one month, participants were invited to a shorter 20-min follow-up survey consisting of PVAQ, CPAQ-R, CPVI, and PASS-20. To ensure data quality, two instructional manipulation checks were embedded in the questionnaire^51,52^. Each question asked participants to select specific numbers on a 4-point scale. Completion times were also monitored, and participants with times outside 2 standard deviations from the mean were excluded (commonly used approach for identifying response time outliers in online surveys^53^). Compensation for participation was provided through “ARIADNA” panel in the form of reward points. The study protocol for the project was preregistered (https://osf.io/aewrc) and received approval from the Local Ethics Committee (KE/20_2023). The research was conducted in compliance with the ethical standards outlined in the 1964 Declaration of Helsinki and its subsequent amendments. All participants have given written informed consent in electronic format. Detailed information on the study procedure is available in a separate publication^37^.

#### Statistical analyses

In the initial phase, we examined descriptive statistics of items and checked the suitability of data for factor analysis. Next, we performed CFA using JASP Version 0.19 to validate the different factor structures identified in previous studies. To account for the ordinal nature and non-normal distribution of the data, we employed the diagonally weighted least squares (DWLS) estimator with robust standard errors^54–56^. The overall fit of the model was assessed using (a) Comparative Fit Index (CFI), (b) Normative Fit Index (NFI), (c) Root Mean Square Error of Approximation (RMSEA) with 90% confidence interval, and (d) chi-square to degrees of freedom ratio (χ^2^/df). A model was considered a good fit if the χ^2^/df ratio was ≤ 2, NFI and CFI were ≥ 0.95, and RMSEA was close to 0.06. A model was considered an acceptable fit if the χ^2^/df ratio was ≤ 5, NFI ≥ 0.90, CFI ≥ 0.90, RMSEA < 0.08^54,57,58^. Additionally, modification indices (MI) were reviewed to identify potentially overlapping items that could be compromising model fit^59^.

Then, we calculated McDonald’s omega (ω) values (< 0.60: poor; 0.60–0.69: marginal; 0.70–0.79: acceptable; > 0.80: good) and item-rest correlations to assess the internal consistency of the scales. Moreover, we evaluated the potential presence of floor and ceiling effects at both the subscales and total score levels. These effects were deemed to exist if more than 15% of participants achieved the minimum or maximum possible score^60^. To evaluate test–retest reliability, the intraclass correlation (ICC) was employed using a two-way random effects model to measure absolute agreement (single rater) in a group of 330 participants. It was interpreted as follows: 0.50–0.75, moderate reliability; 0.75–0.90, good reliability; > 0.90, excellent reliability. The minimal detectable change (MDC) was determined using the formula^61^: 1.96 × SEM × √2, with SEM representing the standard error of measurement. SEM was computed as baseline SD × √1-ICC (SD—standard deviation from the initial measurement).

Correlation analyses were conducted to assess the construct validity of the scale by examining its associations with the DASS-21 depression and anxiety subscales, PASS-20, CSQ Catastrophizing, CAQ-8, SEQ, and pain intensity (both current and over the previous week). The strength of correlation coefficients was interpreted using Cohen’s^62^ guidelines. Bonferroni correction was applied to account for multiple comparisons. For the associations between PVAQ and DASS-21 subscales, PASS-20 total, CSQ, CAQ-8 and SEQ scores, the α-level was adjusted by dividing it by 3 (reflecting the PVAQ total score and two PVAQ subscales), resulting in a significance threshold of *α* < 0.017. For the associations between PVAQ and pain intensity, the significance threshold was adjusted by dividing the α-level by 6 (considering two measures of pain intensity and three PVAQ scores), resulting in a significance threshold of α < 0.008^63,64^.

To assess group-known validity, a series of independent sample t-tests with bootstrapping (5,000 resamples) was conducted. Given that the hypothesis regarding differences in PVAQ scores between disabled and non-disabled individuals involved nine comparisons (three indicators of functional limitations due to pain: sick leave, resignation from work/studies, and resignation from important activities due to pain, and three PVAQ scores), a Bonferroni correction was applied, setting the significance threshold at α < 0.006 for these comparisons.

### Study 2

#### Participants

Since Study 2 began before the final structure of the Polish PVAQ was established, the initial sample size calculation was conducted using the A-Priori Sample Size Calculator for Structural Equation Modelling^65^, assuming the most complex structure of the scale established in the previous studies, including three latent variables and 16 items. This analysis indicated that a minimum sample size of N = 119 was required for adequate model estimation, assuming a medium effect size (0.30). The final collected sample comprised 148 participants, including 33 men (22.30%), 114 women (77.03%), and one individual with unspecified gender (0.68%), with ages ranging from 18 to 85 years (M = 56.56, SD = 16.33).

Participants were recruited from the Pain Research and Treatment Unit of the University Hospital in Krakow. The eligibility criteria were identical to Study 1. All participants provided written informed consent to take part in the study and did not receive any financial compensation for their involvement. The research was conducted in compliance with the ethical standards outlined in the 1964 Declaration of Helsinki. The study protocol was approved by the Local Ethics Committee (KE/20_2023) Detailed participant characteristics are described elsewhere^37^.

#### Measures and procedure

Similarly to Study 1, participants were asked to complete a preliminary survey and a set of questionnaires: PVAQ (full 16-item version), CPAQ, PASS-20 and CPVI.

The data were collected from September to December 2023. Patients were recruited by researchers during their visits to the pain treatment facility, either completing the paper-based survey on-site or accessing the online version via the provided Quick Response (QR) Code. In total, 131 paper surveys and 17 online responses were obtained (see:^37^ for details).

#### Statistical analyses

In the initial step, multivariate normality as well as floor and ceilings effects were assessed. Then, a CFA (using the DWLS estimator) was performed to evaluate the two-factor structure of the Polish PVAQ-12 obtained in Study 1. CFI, NFI, RMSEA, χ^2^/df were utilized to examine model fit. McDonald’s Omega was calculated as a measure of internal consistency.

Correlation analysis was conducted to examine the relationship between PVAQ-12 and pain intensity. Consistent with Study 1, the significance threshold was adjusted by dividing the α-level by 6. Independent t-tests (with Bootstrap, *N* = 5000) were performed to explore differences in the total PVAQ-12 score between individuals who were on sick leave due to pain and those who were not, between those who reported having left their job or university due to pain and those who had not, and between those who reported having to give up or limit activities important to them and those who had not (known-group validity). Since the hypothesis regarding differences in PVAQ-12 scores between disabled and non-disabled individuals was tested across nine comparisons (three indicators of functional limitations due to pain and three PVAQ-12 scores), Bonferroni correction was applied, setting a significance threshold of *α* < 0.006. Cases with missing data were deleted listwise.

## Supplementary Information

Supplementary Information.

## Data Availability

The datasets analyzed in the current study are available in the RODBUK repository (https://doi.org/10.57903/UJ/VOPA3J).
