# Supplementary material for: Development and psychometric evaluation of the 12-item Polish version of the Pain Vigilance and Awareness Questionnaire
Source: Sci Rep. 2025 Jul 22;15:26541. doi: 10.1038/s41598-025-08449-6 (PMC12280029; doi:10.1038/s41598-025-08449-6)
Supplement: Supplementary file 1 — Supplementary Information. [file 41598_2025_8449_MOESM1_ESM.docx]

**Supplementary materials**

Kłosowska, J., Budzisz, A., Rubanets, D., Łaska, I. A., Bieniek, H., Brączyk, J., Wiercioch-Kuzianik, K., Bajcar, E. A., Żegleń, M., Niedbał, M., Badzińska, J., Przeklasa-Muszyńska, A., McCracken M. L., Bąbel, P. (2025). Development and Psychometric Evaluation of the 12-Item Polish Version of the Pain Vigilance and Awareness Questionnaire. *Scientific Reports*

Table of contents:

[**Supplementary Table 1.** Descriptive statistics for PVAQ items – Study 1 2](#_Toc200189693)

[**Supplementary Table 2.** Fit indexes of the tested models 3](#_Toc200189694)

[**Supplementary Table 3.** Results of Exploratory Factor Analysis – Study 1 4](#_Toc200189695)

[**Supplementary Table 4.** Item-rest correlations (total scale) calculated for the original 16-item PVAQ – Study 1 6](#_Toc200189696)

[**Supplementary Table 5.** Results of Confirmatory Factor Analysis – Study 1 (online sample) 7](#_Toc200189697)

[**Supplementary Table 6.** Item reliability statistics – Study 1 8](#_Toc200189698)

[**Supplementary Table 7.** Correlations (Pearson) of PVAQ-12 with other scales – Study 1 9](#_Toc200189699)

[**Supplementary Table 8.** Descriptive statistics for PVAQ items and subscales – Study 2 (hospital-recruited sample) 10](#_Toc200189700)

[**Supplementary Table 9.** Item reliability statistics – Study 2 (hospital-recruited sample) 11](#_Toc200189701)

[**Supplementary Table 10.** Comparison of item allocations to Active Vigilance and Passive Awareness subscales of PVAQ in previous research and the current study 12](#_Toc200189702)

[**Polish version of the PVAQ** 13](#_Toc200189703)

## **Supplementary Table 1.** Descriptive statistics for PVAQ items – Study 1

|  | **Total sample** | | **Women** | | **Men** | | **Total sample** | | |
| --- | --- | --- | --- | --- | --- | --- | --- | --- | --- |
|  | ***Mean*** | ***SD*** | ***Mean*** | ***SD*** | ***Mean*** | ***SD*** | ***S*** | ***K*** | **Min./Max.** |
| *Measurement 1* | | | | | | | | | |
| Item 1 | 2.79 | 1.28 | 2.9 | 1.27 | 2.63 | 1.28 | -0.04 | -0.77 | 0/5 |
| Item 2 | 3.54 | 1.06 | 3.6 | 1.02 | 3.44 | 1.11 | -0.52 | 0.13 | 0/5 |
| Item 3 | 3.68 | 1 | 3.7 | 0.97 | 3.64 | 1.04 | -0.6 | 0.44 | 0/5 |
| Item 4 | 3.4 | 1.11 | 3.47 | 1.08 | 3.31 | 1.15 | -0.45 | -0.07 | 0/5 |
| Item 5 | 3.57 | 1.03 | 3.59 | 1 | 3.54 | 1.08 | -0.48 | -0.05 | 0/5 |
| Item 6 | 3.04 | 1.22 | 3.08 | 1.2 | 2.98 | 1.25 | -0.26 | -0.47 | 0/5 |
| Item 7 | 3.62 | 1.08 | 3.73 | 1.03 | 3.45 | 1.12 | -0.47 | -0.27 | 0/5 |
| Item 8 | 2.3 | 1.25 | 2.12 | 1.28 | 2.57 | 1.17 | 0.08 | -0.57 | 0/5 |
| Item 9 | 3.39 | 1.09 | 3.38 | 1.15 | 3.42 | 0.98 | -0.44 | 0.04 | 0/5 |
| Item 10 | 2.83 | 1.27 | 2.82 | 1.31 | 2.84 | 1.21 | -0.30 | -0.44 | 0/5 |
| Item 11 | 3.32 | 1.14 | 3.27 | 1.17 | 3.39 | 1.11 | -0.27 | -0.34 | 0/5 |
| Item 12 | 2.96 | 1.35 | 2.96 | 1.41 | 2.95 | 1.27 | -0.4 | -0.44 | 0/5 |
| Item 13 | 2.53 | 1.34 | 2.48 | 1.36 | 2.6 | 1.31 | -0.02 | -0.65 | 0/5 |
| Item 14 | 2.78 | 1.34 | 2.78 | 1.35 | 2.79 | 1.33 | -0.15 | -0.73 | 0/5 |
| Item 15 | 2.64 | 1.38 | 2.73 | 1.42 | 2.51 | 1.32 | 0.01 | -0.78 | 0/5 |
| Item 16 | 2.58 | 1.33 | 2.43 | 1.35 | 2.79 | 1.29 | -0.09 | -0.76 | 0/5 |
| *Measurement 2* | | | | | | | | | |
| Item 1 | 2.84 | 1.24 | 3 | 1.2 | 2.63 | 1.27 | -0.01 | -0.64 | 0/5 |
| Item 2 | 3.33 | 1.08 | 3.39 | 1.04 | 3.24 | 1.12 | -0.45 | 0.13 | 0/5 |
| Item 3 | 3.46 | 1.09 | 3.55 | 1.05 | 3.33 | 1.15 | -0.54 | 0.06 | 0/5 |
| Item 4 | 3.28 | 1.22 | 3.42 | 1.14 | 3.1 | 1.29 | -0.41 | -0.25 | 0/5 |
| Item 5 | 3.36 | 1.11 | 3.46 | 1.04 | 3.24 | 1.19 | -0.48 | 0.03 | 0/5 |
| Item 6 | 2.98 | 1.26 | 3.13 | 1.23 | 2.79 | 1.26 | -0.2 | -0.58 | 0/5 |
| Item 7 | 3.38 | 1.13 | 3.57 | 1.06 | 3.14 | 1.18 | -0.51 | 0.06 | 0/5 |
| Item 8 | 2.28 | 1.22 | 2.18 | 1.19 | 2.42 | 1.26 | 0.01 | -0.53 | 0/5 |
| Item 9 | 3.19 | 1.21 | 3.28 | 1.19 | 3.08 | 1.23 | -0.37 | -0.30 | 0/5 |
| Item 10 | 2.74 | 1.26 | 2.66 | 1.27 | 2.84 | 1.24 | -0.31 | -0.44 | 0/5 |
| Item 11 | 3.16 | 1.18 | 3.22 | 1.16 | 3.08 | 1.22 | -0.28 | -0.37 | 0/5 |
| Item 12 | 2.82 | 1.28 | 2.94 | 1.28 | 2.67 | 1.26 | -0.3 | -0.37 | 0/5 |
| Item 13 | 2.52 | 1.27 | 2.57 | 1.33 | 2.46 | 1.19 | 0.03 | -0.47 | 0/5 |
| Item 14 | 2.72 | 1.26 | 2.74 | 1.3 | 2.69 | 1.21 | -0.13 | -0.42 | 0/5 |
| Item 15 | 2.56 | 1.28 | 2.68 | 1.33 | 2.41 | 1.2 | 0.04 | -0.57 | 0/5 |
| Item 16 | 2.51 | 1.3 | 2.48 | 1.3 | 2.54 | 1.29 | -0.14 | -0.82 | 0/5 |

*Note: Measurement 1: N=418, Measurement 2: N=330; S* – *Skewness; K* – *Kurtosis*

## **Supplementary Table 2.** Fit indexes of the tested models

| **Model** | **Study** | **No. Factors** | **No. items** | **Deleted items** | ***χ2*** | ***df*** | ***χ2/df*** | ***NFI*** | ***CFI*** | ***RMSEA***  **(90% *CI)*** |
| --- | --- | --- | --- | --- | --- | --- | --- | --- | --- | --- |
| M1 | McCracken, 1997 | 1 | 16 | - | 989.50 | 104 | 9.51 | .90 | .91 | .14 (.14 - .15) |
| M2 | McWilliams & Asmundson (2001) | 3 | 16 | - | 556.40 | 101 | 5.51 | .94 | .95 | .10 (.10 - .11) |
| M3 | Roelofs, 2002; Kunz et al., 2017 | 2 | 16 | - | 762.56 | 103 | 7.43 | .92 | .93 | .12 (.12 - .13) |
| M4 | Roelofs, 2003 | 2 | 14 | 8,16 | 583.02 | 76 | 7.67 | .94 | .95 | .13(.12, .14) |
| M5 | McCracken, 2007; Wong, 2011; Monticone, 2016 | 2 | 13 | 2, 8, 16 | 355.72 | 64 | 5.56 | .96 | .96 | .11(.09, .12) |
| M6 | Esteve, 2013; Martinez, 2014 | 2 | 9 | 1, 4, 7, 8, 10, 15, 16 | 199.65 | 26 | 7.68 | .95 | .96 | .13(.11, .14) |
| M7 | Bonafe, 2018 | 2 | 13 | 1, 8, 16 | 539.95 | 64 | 8.44 | .94 | .95 | .13 (.12, .14) |
| M8 | Hoffmann, 2023 | 2 | 8 | 1, 4, 7, 8, 10, 11, 15, 16 | 143.46 | 19 | 7.55 | .96 | .97 | .13(.11, .15) |

*Note: N=418; M – Model*

## **Supplementary Table 3.** Results of Exploratory Factor Analysis – Study 1

| **PVAQ Item** | **Factor 1** | **Factor 2** | **Factor 3** |
| --- | --- | --- | --- |
| 16-items |  |  |  |
| Item 2 | 0.908 |  |  |
| Item 3 | 0.902 |  |  |
| Item 5 | 0.887 |  |  |
| Item 7 | 0.809 |  |  |
| Item 11 | 0.716 |  |  |
| Item 9 | 0.68 |  |  |
| Item 4 | 0.601 |  |  |
| Item 10 | 0.388 | 0.424 |  |
| Item 13 |  | 0.983 |  |
| Item 14 |  | 0.943 |  |
| Item 15 |  | 0.915 |  |
| Item 12 |  | 0.580 |  |
| Item 6 |  | 0.517 |  |
| Item 1 |  | 0.374 | -0.331 |
| Item 8 |  |  | 0.767 |
| Item 16 |  |  | 0.564 |
| Variance | 29% | 23% | 9% |
| 14-items |  |  |  |
| Item 2 | 0.894 |  |  |
| Item 3 | 0.889 |  |  |
| Item 5 | 0.869 |  |  |
| Item 7 | 0.795 |  |  |
| Item 11 | 0.700 |  |  |
| Item 9 | 0.668 |  |  |
| Item 4 | 0.599 |  |  |
| Item 13 |  | 0.969 |  |
| Item 14 |  | 0.938 |  |
| Item 15 |  | 0.886 |  |
| Item 12 |  | 0.567 |  |
| Item 6 |  | 0.503 |  |
| Item 8 |  |  | 0.793 |
| Item 16 |  |  | 0.637 |
| Variance | 31% | 23% | 9% |
| 12-items |  |  |  |
| Item 3 | 0.890 |  |  |
| Item 2 | 0.888 |  |  |
| Item 5 | 0.870 |  |  |
| Item 7 | 0.779 |  |  |
| Item 11 | 0.700 |  |  |
| Item 9 | 0.670 |  |  |
| Item 4 | 0.595 |  |  |
| Item 13 |  | 0.974 |  |
| Item 14 |  | 0.945 |  |
| Item 15 |  | 0.898 |  |
| Item 12 |  | 0.539 |  |
| Item 6 |  | 0.512 |  |
| Variance | 36% | 27% |  |

*Note: N=209; Only factor loadings > .30 are presented; Principal Component Analysis conducted on polychronic correlations; Rotation method: Promax*

## **Supplementary Table 4.** Item-rest correlations (total scale) calculated for the original 16-item PVAQ – Study 1

|  | **If item dropped** |  |
| --- | --- | --- |
| **Item** | **McDonald’s *ω*** | **Item-rest correlation** |
| Item 1 | 0.873 | 0.475 |
| Item 2 | 0.87 | 0.573 |
| Item 3 | 0.868 | 0.634 |
| Item 4 | 0.874 | 0.483 |
| Item 5 | 0.869 | 0.629 |
| Item 6 | 0.864 | 0.655 |
| Item 7 | 0.866 | 0.646 |
| **Item 8** | **0.896** | **-0.173** |
| Item 9 | 0.871 | 0.560 |
| Item 10 | 0.867 | 0.619 |
| Item 11 | 0.873 | 0.513 |
| Item 12 | 0.869 | 0.591 |
| Item 13 | 0.863 | 0.660 |
| Item 14 | 0.861 | 0.691 |
| Item 15 | 0.864 | 0.639 |
| **Item 16** | **0.895** | **-0.018** |

*Note N=209*

## **Supplementary Table 5.** Results of Confirmatory Factor Analysis – Study 1 (online sample)

|  | | | | | | **95% Confidence Interval** | |
| --- | --- | --- | --- | --- | --- | --- | --- |
| **Factor** | **Indicator** | **Estimate** | ***SE*** | ***z*-value** | ***p*** | **Lower** | **Upper** |
| Factor 1  Passive Awareness | Item 2 | .644 | 0.044 | 14.776 | <.001 | .559 | .730 |
|  | Item 3 | .681 | .038 | 18.062 | <.001 | .607 | .755 |
|  | Item 4 | .652 | .041 | 15.961 | <.001 | .572 | .733 |
|  | Item 5 | .806 | .033 | 24.663 | <.001 | .742 | .870 |
|  | Item 7 | .744 | .041 | 18.068 | <.001 | .663 | .825 |
|  | Item 9 | .571 | .048 | 11.989 | <.001 | .478 | .665 |
|  | Item 11 | .552 | .049 | 11.225 | <.001 | .456 | .648 |
| Factor 2  Active Vigilance | Item 6 | .830 | .030 | 27.868 | <.001 | .772 | .888 |
|  | Item 12 | .579 | .047 | 12.287 | <.001 | .487 | .672 |
|  | Item 13 | .682 | .039 | 17.406 | <.001 | .605 | .759 |
|  | Item 14 | .743 | .034 | 21.725 | <.001 | .676 | .810 |
|  | Item 15 | .738 | .035 | 20.847 | <.001 | .668 | .807 |

*Note: N=209*

## **Supplementary Table 6.** Item reliability statistics – Study 1

|  | **If item dropped** | | **Item-rest correlation** | |
| --- | --- | --- | --- | --- |
| **Item** | **Subscale *ω*** | **Total *ω*** | **Subscale** | **Total** |
| Factor 1: Passive Awareness |  |  |  |  |
| Item 2 | 0.858 | 0.892 | 0.679 | 0.572 |
| Item 3 | 0.852 | 0.889 | 0.731 | 0.641 |
| Item 4 | 0.874 | 0.895 | 0.546 | 0.501 |
| Item 5 | 0.853 | 0.890 | 0.717 | 0.633 |
| Item 7 | 0.859 | 0.889 | 0.663 | 0.645 |
| Item 9 | 0.861 | 0.889 | 0.654 | 0.646 |
| Item 11 | 0.864 | 0.891 | 0.639 | 0.592 |
| Factor 2: Active Vigilance |  |  |  |  |
| Item 6 | 0.865 | 0.887 | 0.631 | 0.643 |
| Item 12 | 0.888 | 0.893 | 0.524 | 0.561 |
| Item 13 | 0.828 | 0.887 | 0.806 | 0.647 |
| Item 14 | 0.830 | 0.885 | 0.791 | 0.675 |
| Item 15 | 0.842 | 0.889 | 0.743 | 0.610 |

*Note: N=418*

## **Supplementary Table 7.** Correlations (Pearson) of PVAQ-12 with other scales – Study 1

| **Variable** | PVAQ-12  Total score | | .PVAQ-12  Passive awareness | | PVAQ-12  Active vigilance | |
| --- | --- | --- | --- | --- | --- | --- |
| DASS-21 Depression | .15 | ** | .06 |  | .20 | *** |
| DASS-21 Anxiety | .22 | *** | .08 |  | .30 | *** |
| CSQ Catastrophizing | .39 | *** | .18 | *** | .51 | *** |
| PASS-20 Total score | .54 | *** | .35 | *** | .60 | *** |
| CAQ-8 | .04 |  | .20 | *** | -.13 | ** |
| SEQ | .21 | *** | .33 | *** | .05 |  |
| Average pain - now | .22 | *** | .15 | ** | .23 | *** |
| Average pain - previous week | .27 | *** | .29 | *** | .19 | *** |

*Note: *p<.05, **p<.01; ***p<.001, # did not survive adjustment for multiple comparisons; N=418; PVAQ-12 – Pain Vigilance and Awareness Scale 12-item version; DASS-21 – Depression Anxiety Stress Scales 21-item version; CSQ – Coping Strategies Questionnaire; PASS-20 – Pain Anxiety Symptom Scale 20-item version; CAQ-8 – 8-item version of Committed Action Questionnaire; SEQ – Self Experiences Questionnaire*

## **Supplementary Table 8.** Descriptive statistics for PVAQ items and subscales – Study 2 (hospital-recruited sample)

| **PVAQ-12 Item** | ***N*** | ***Mean*** | ***SD*** | ***S*** | ***K*** | **Min./Max.** |
| --- | --- | --- | --- | --- | --- | --- |
| Item 2 | 138 | 3.928 | 1.098 | -1.364 | 1.995 | 0-5 |
| Item 3 | 138 | 3.957 | 1.066 | -1.269 | 1.667 | 0-5 |
| Item 4 | 138 | 3.500 | 1.410 | -0.848 | -.192 | 0-5 |
| Item 5 | 137 | 3.825 | 1.212 | -1.292 | 1.501 | 0-5 |
| Item 6 | 138 | 3.225 | 1.490 | -0.353 | -.930 | 0-5 |
| Item 7 | 138 | 3.986 | 1.196 | -1.297 | 1.489 | 0-5 |
| Item 9 | 136 | 3.426 | 1.499 | -0.975 | .016 | 0-5 |
| Item 11 | 135 | 3.215 | 1.483 | -0.669 | -.477 | 0-5 |
| Item 12 | 137 | 3.022 | 1.560 | -0.473 | -.758 | 0-5 |
| Item 13 | 137 | 2.540 | 1.510 | 0.039 | -.943 | 0-5 |
| Item 14 | 136 | 2.750 | 1.623 | -0.326 | -1.061 | 0-5 |
| Item 15 | 137 | 2.876 | 1.611 | -0.267 | -1.058 | 0-5 |
| Passive Awareness | 132 | 25.871 | 6.373 | -1.397 | 2.841 | 0-35 |
| Active Vigilance | 135 | 14.452 | 6.011 | -.190 | -.582 | 0-25 |
| Total score | 131 | 40.153 | 11.065 | -.803 | 1.059 | 1 - 60 |

**Note:** PVAQ-12 – *Pain Vigilance and Awareness Scale 12-item version; S* – *Skewness, K* – *Kurtosis*

## **Supplementary Table 9.** Item reliability statistics – Study 2 (hospital-recruited sample)

|  | **If item dropped** | | **Item-rest correlation** | |
| --- | --- | --- | --- | --- |
| **Item** | ***ω* Subscale** | ***ω* Total** | **Subscale** | **Total** |
| Passive Awareness |  |  |  |  |
| Item 2 | .821 | .877 | .585 | .532 |
| Item 3 | .806 | .872 | .719 | .689 |
| Item 4 | .832 | .882 | .535 | .460 |
| Item 5 | .797 | .871 | .735 | .675 |
| Item 7 | .798 | .868 | .738 | .729 |
| Item 9 | .838 | .877 | .493 | .545 |
| Item 11 | .845 | .884 | .450 | .425 |
| Active Vigilance |  |  |  |  |
| Item 6 | .795 | .865 | .695 | .719 |
| Item 12 | .858 | .882 | .414 | .462 |
| Item 13 | .771 | .871 | .763 | .626 |
| Item 14 | .805 | .873 | .647 | .603 |
| Item 15 | .802 | .873 | .660 | .599 |

## **Supplementary Table 10.** Comparison of item allocations to Active Vigilance and Passive Awareness subscales of PVAQ in previous research and the current study

| Study | Item number (according to the original 16-item PVAQ) | | | | | | | | | | | | | | | |
| --- | --- | --- | --- | --- | --- | --- | --- | --- | --- | --- | --- | --- | --- | --- | --- | --- |
|  | 1 | 2 | 3 | 4 | 5 | 6 | 7 | 8 | 9 | 10 | 11 | 12 | 13 | 14 | 15 | 16 |
| McCracken, 2007; Wong et al., 2011; Monticone et al., 2016 | A |  | A | A | A | V | A |  | A | V | A | V | V | V | V |  |
| Esteve et. al., 2013; Martinez et al., 2014 |  | A | A |  | A | V |  |  | A |  | A | V | V | V |  |  |
| Hoffmann et al., 2024 |  | A | A |  | A | V |  |  | A |  |  | V | V | V |  |  |
| Current study |  | A | A | A | A | V | A |  | A |  | A | V | V | V | V |  |

*Note: A – Passive Awareness subscale, V – Active Vigilance subscale of Pain Vigilance and Awareness Questionnaire (PVAQ)*

## **Polish version of the PVAQ**

**Kwestionariusz czujności i świadomości bólu – wersja polska (PVAQ-12 PL)**

Poniżej znajduje się 12 opisów, jak ludzie reagują na ból. Za pomocą poniższej skali prosimy wskazać, jak często dane stwierdzenie ma zastosowanie w Pana/_/Pani przypadku (tj. jak często reaguje Pan/_/Pani w opisany sposób). W ocenie prosimy wziąć pod uwagę ostatnie dwa tygodnie. W każdym punkcie prosimy zakreślić właściwą cyfrę od 0 (nigdy) do 5 (zawsze).

Nigdy Zawsze

1. (2) Mam świadomość nagłych lub chwilowych zmian bólu 0 1 2 3 4 5
2. (3) Szybko zauważam zmiany w natężeniu bólu 0 1 2 3 4 5
3. (4) Szybko zauważam wpływ leków na odczuwany przeze mnie ból 0 1 2 3 4 5
4. (5) Szybko zauważam zmiany umiejscowienia lub zakresu bólu 0 1 2 3 4 5
5. (6) Skupiam uwagę na dolegliwościach bólowych 0 1 2 3 4 5
6. (7) Zauważam ból, nawet jeśli wykonuję akurat jakąś czynność 0 1 2 3 4 5
7. (9) Od razu wiem, kiedy ból nadchodzi albo narasta. 0 1 2 3 4 5
8. (11) Od razu wiem, kiedy ból słabnie. 0 1 2 3 4 5
9. (12) Wydaje mi się, że mam większą świadomość bólu niż inni 0 1 2 3 4 5
10. (13) Poświęcam swojemu bólowi dużo uwagi 0 1 2 3 4 5
11. (14) Śledzę natężenie swojego bólu 0 1 2 3 4 5
12. (15) Ból bardzo mnie absorbuje 0 1 2 3 4 5

© 2025 The Authors. All Rights Reserved.

*(Item numbers in brackets refer to the original PVAQ items as published in the article by Lance M. McCracken (1997). This article was published in Behavior Therapy, Volume 28, Issue 2, Lance M. McCracken, “Attention” to pain in persons with chronic pain A behavioral approach, Pages 271-284, Copyright Elsevier (1997)).*
